# Real-World Evidence of the Impact of a Novel Surgical Irrigant on Surgical Site Infections in Primary Total Knee Arthroplasty Performed at an Ambulatory Surgery Center

**DOI:** 10.1089/sur.2023.304

**Published:** 2024-04-04

**Authors:** Ronald W. Singer

**Affiliations:** ^1^ Edgewater Surgery Center, Fort Mill, South Carolina, USA.; ^2^ OrthoCarolina, Charlotte, North Carolina, USA.

**Keywords:** knee arthroplasty, surgical site infection, XPERIENCE

## Abstract

**Background:**

Total knee arthroplasty (TKA) is one of the most common inpatient and outpatient surgical procedures performed in the United States and is predicted to increase 401% by 2040. Surgical site infections (SSIs) at an incidence rate of approximately 2% are costly post-operative complications in TKA. Intra-operative surgical irrigants are used to decrease contaminating microbial bioburden within the surgical site to prevent SSI. The primary objective of this retrospective study was to evaluate the impact of a novel surgical irrigant called XPERIENCE^®^ Advanced Surgical Irrigation (XP; Next Science, Jacksonville, FL) on SSI incidence in primary TKA performed at an ambulatory surgery center (ASC).

**Patients and Methods:**

Primary TKAs were performed at a free-standing ASC. The novel surgical irrigant was used intra-operatively to rinse away debris and micro-organisms from the surgical site. Retrospective data collation included SSI rates, complication rates, and re-admissions due to SSI within 90 days of index surgery.

**Results:**

Among the 524 primary TKA surgeries, one peri-prosthetic joint infection (PJI) was diagnosed within 90 days of index surgery and one superficial incisional SSI was diagnosed within 30 days of index surgery. The PJI was attributed to an exogenously acquired upper respiratory tract infection rather than due to the failure of intra-operative regimes. The 0.19% PJI incidence rate indicated significant efficacy of XP in decreasing PJI. An overall complication rate of 7.82% was noted with none of the complications associated with usage of the novel surgical irrigant.

**Conclusions:**

XPERIENCE is a promising intra-operative antimicrobial irrigant that can be easily incorporated into a broader infection prevention strategy.

Primary total knee arthroplasty (TKA) is one of the most common elective surgical procedures performed in the United States.^1,2^ Compared with 2014 the annual number of TKAs are predicted to increase by 401% in 2040 to 3,416,000 replacements.^3^ With TKA procedures being removed from Medicare's Inpatient-Only List effective January 2018, TKA procedures are now in the top 20 surgical procedures performed on an outpatient basis.^2^ Approximately 36% of all TKA were performed on an outpatient basis in 2019 compared with 0.2% in 2017.^4^ Although outpatient TKA surgeries afford a level of perceived convenience and cost savings, differing patient profiles such as age and comorbidities can impact outcomes in outpatient versus inpatient settings.^5–8^ The scientific literature has contradictory narratives, ranging from lower, similar, or higher SSI and complication rates in outpatient/ambulatory versus inpatient TKAs.^9–15^

The U.S. Centers for Disease Control and Prevention (CDC) defines SSI as an infection that occurs after surgery in the part of the body where the surgery took place. The CDC's National Health Safety Network (NHSN) has defined criteria for diagnosing SSI in joint replacement surgery at three levels: superficial incisional SSI, deep incisional SSI, and organ/space SSI/early onset peri-prosthetic joint infection (PJI).^16^ Superficial incisional SSI occurring within 30 days of index surgery typically involves only the skin and subcutaneous tissue. Deep incisional SSI occurring within 90 days post-index surgery involves deep soft tissues such as fascia and muscle layers. Organ/space SSI/ early onset PJI refers to an infection occurring in any part of the anatomy other than the incision, which has been opened or manipulated during the operative procedure within 90 days of index surgery and includes the implant.^16^ Surgical site infections can occur in approximately 2% of primary TKA surgeries and are considered serious complications leading to high patient morbidity, mortality, and economic burden, with PJI being the most common cause for revision associated with failed knee replacement.^17–20^

According to the National Institutes of Health (NIH), 80% of microbial infections in the body are attributable to the formation of biofilms, which are also implicated in SSI and prosthesis-related infections after joint replacements.^21–23^ Biofilms are complex microbial communities encased within a protective matrix of self-produced extracellular polymeric substances (EPS) strengthened with metal ion crosslinks, that can attach to biotic and abiotic surfaces.^22–24^ Mature biofilms exhibit intrinsic recalcitrance to antibiotic agents, antimicrobial agents, and host immune defenses making their eradication particularly difficult.^24–27^ Thus, it is imperative to prevent the formation of biofilms to obviate peri- and post-operative SSI/PJI by instituting a multitiered infection prevention approach, including the use of innovative intra-operative irrigants to decrease contaminating microbial bioburden within the surgical incision space.^28–31^

## Patients and Methods

### Setting and procedures

Primary TKAs were performed between May 2021, and May 2023, at Edgewater Surgery Center, a free-standing ambulatory surgery center (ASC) in Fort Mill, South Carolina. All surgeries were performed by a single orthopedic surgeon using patient specific instrumentation (Visionaire technique: Smith and Nephew, Memphis, TN) in most of the surgeries whereas traditional instrumentation techniques were used in approximately 1% of the surgeries. XPERIENCE^®^ Advanced Surgical Irrigation (XP; Next Science, Jacksonville, FL), a novel antimicrobial surgical irrigant was flushed in the surgical incision space via pulsed lavage. The novel surgical irrigant was used prior to cementation, mechanically agitated in the surgical area, and then suctioned after which cancellous bone surfaces were carefully dried. It was subsequently used after cementation, just prior to closure. No other irrigant was used after the administration of XP. A maximum volume of 500 mL of the novel surgical irrigant was used intra-operatively during the entire procedure and excess solution was suctioned away without rinsing. Antibiotic beads or powder were not used intra-operatively. All patients were discharged to home on the day of surgery with most patients being discharged within three to four hours after surgery.

XPERIENCE consists of citric acid, sodium citrate, and sodium lauryl sulfate in water. Citric acid and sodium citrate serve as pH buffers and aid in metal ion chelation, while sodium lauryl sulfate acts as a surfactant, assisting in debris removal including micro-organisms.

### Data collection

An Institutional Review Board (IRB) exemption was granted for the study under 45 CFR § 46.104(d)(4) based on the use of identifiable subject information that was recorded by the principal investigator in a manner that the subject's identity could not be readily ascertained directly or through identifiers linked to the patient. The principal investigator conducted a systematic evaluation of the surgery schedule at Edgewater between May 2021 and May 2023. Subjects were selected for inclusion in the retrospective analysis based on defined inclusion/exclusion criteria.

Inclusion criteria included:

- Surgery concerned primary TKA where the complete native knee joint was replaced with an implant.
- Subjects were treated intra-operatively with XP.
- Subjects had signed a surgical consent form, which did not prohibit use of protected health information.
- Subjects had follow-up care for at least 90 days post-index surgery.
- Subjects were 18 years and older.

Exclusion criteria included:

- Subjects were from a vulnerable population in accordance with 45 CFR 46 Subparts B, C, and D.
- Subjects who exhibited signs or symptoms of infection at the time of surgery.
- Subjects who were administered antibiotic beads during surgery.
- Subjects who required additional surgical intervention unrelated to primary TKA surgery within 90 days of index surgery.

### Clinical study end points

The primary end point for the retrospective study was the incidence of subjects diagnosed with deep incisional or organ space SSI/PJI within 90 days of the index surgery whereas the secondary end point was the incidence of subjects diagnosed with superficial incisional SSI within 30 days of the index surgery following the CDC's NHSN criteria for diagnosis of SSI.

The exploratory and safety end points for the study were:

- Incidences of re-admission because of SSI within 90 days of index surgery.
- Incidences of peri-operative QT prolongation in subjects given XP, as captured via standard peri-operative electrocardiographic monitoring.
- Incidences of adverse events or complications experienced within 90 days post-index surgery.

Only the principal investigator with documented access to the Epic, Allscripts, and SIS databases accessed data relevant for this retrospective data compilation. The databases included admission records, medical charts, pre-operative, operative, recovery room notes, anesthesia notes, cardiac readouts, post-operative follow-up records, re-admission records, and other relevant documentation.

The data mining occurred between July 2022 and August 2023 in accordance with the institution's guidelines and collated into a secure Excel spreadsheet (Microsoft, Redmond, WA). The Excel data was validated by confirming 100% of the source data. Collated data included:

- Subject initials.
- Date of surgery.
- Type of surgery.
- Diagnosis of deep incisional SSI, organ/space SSI/early onset PJI within 90 days of index surgery using NHSN criteria.
- Diagnosis of superficial incisional SSI within 30 days of index surgery using NHSN criteria.
- Re-admissions due to SSI/PJI within 90 days of index surgery.
- Any change to cardiac profile during or after surgery.
- Adverse events and complications within 90 days of index surgery.

### Statistical analysis

A two-tailed exact binomial test was used to compare the SSI/PJI rates against historical reported rates and estimate 95% confidence interval of the current study cohort.

## Results

A total of 525 consecutive primary TKA surgeries were performed at the ASC from May 2021 to May 2023 where the novel surgical irrigant was used intra-operatively. Five hundred twenty-four of those surgeries met the inclusion/exclusion criteria for evaluation in the study. Thirty-one subjects underwent sequential bilateral primary TKAs during the study observation period and the sequential surgery was included as a separate primary TKA index surgery. Based on screening for appropriateness for ambulatory TKA surgery by the anesthesia surgical team and the institution's pre-operative surgical criteria, patients typically had American Society of Anesthesiologists (ASA) score ≥III, body mass index under 40, and hemoglobin A1C under 8. Contraindications for surgery at the ASC included patients with cardiac, pulmonary, hepatic, or renal disease. All surgeries included in the study had a minimum of 90 days of follow-up.

Primary end point SSI/PJI assessment within 90 days post-index surgery established that one of the 524 primary TKA surgeries was diagnosed with an early onset PJI at 36 days post-index surgery and *Streptococcus pyogenes* was identified as the causative agent. The PJI diagnosis was preceded by a diagnosis of an upper respiratory tract group A streptococcal pharyngitis attributed to an exogenous exposure several days after the surgery. The development of the PJI was therefore not associated with failure of intra-operative infection prevention regimes. The subject underwent irrigation and debridement with polyethylene exchange and was treated with intravenous and oral cephalosporin antibiotic agents.

The secondary end point evaluations for superficial incisional SSI within 30 days of index surgery determined that a single subject met all the required criteria as delineated by NHSN criteria.^16^ The subject underwent an incision and drainage procedure with drain placed at six weeks post-index surgery and the superficial SSI resolved uneventfully. There was no deep tissue involvement identified. There were no diagnoses of deep incisional SSI in the cohort. Thus, an overall PJI incidence rate of approximately 0.19% (1/524) was noted within 90 days post-index surgery. To compare infection incidence rates in this retrospective study with historical reported rates, a non-expansive review of the peer reviewed literature was conducted. Table 1 lists infection incidences in XP-treated primary TKA in this study compared with relevant literature-reported ranges. In every instance, the use of the novel surgical irrigant resulted in SSI/PJI rates that were consistently below or at the lower range of historical reported rates, indicating a potentially enhanced capability to decrease contaminating bioburden intra-operatively, possibly resulting in a decrease in SSI/PJI incidence compared with literature-reported rates.

**Table 1.** Infection Incidence in XPERIENCE-Treated Primary TKA Cohort versus Historical Reported Rates

Infection description	XPERIENCE incidence rate [95% CI]^a^	Historical incidence rate	*p* ^a^	Historical incidence reference(s)
*Superficial Incisional SSI*	*0.19%*	*0.82%*	*0.21*	*https://pubmed.ncbi.nlm.nih.gov/36726345*
	*[<0.001%–1.1%]*	*3.1%*	*< 0.001*	*https://pubmed.ncbi.nlm.nih.gov/32253138*
Deep Incisional SSI	0.00% [0%–0.073%]	0.65%	0.086	https://pubmed.ncbi.nlm.nih.gov/36726345
PJI	0.19%	0.15%–0.97%	0.53–0.10	https://pubmed.ncbi.nlm.nih.gov/21550765
	[<0.001%–1.1%]	2.18%	< 0.001	https://pubmed.ncbi.nlm.nih.gov/22554729

CI = confidence interval; TKA = total knee arthroplasty.

^a^ 95% CI and p value estimated using exact binomial test.

The exploratory end point evaluating re-admissions attributable to an SSI diagnosis within 90 days of index surgery determined that there were two re-admissions attributable to SSI diagnoses. The exploratory end point evaluating changes in cardiac profile during and after surgery, including peri-operative QT prolongation, as captured via standard peri-operative electrocardiographic monitoring determined that none of the subjects exhibited any adverse changes in cardiac profile when the novel surgical irrigant was used intra-operatively.

A detailed evaluation of complications/adverse events for all 524 subjects was conducted (Table 2). A total of 40 subjects experienced complications/adverse events within 90 days post-index surgery. Thirty-nine subjects experienced a single complication/adverse event whereas one subject experienced dual complications of arthrofibrosis requiring arthroscopy and traumatic medial retinacular rupture/patellar subluxation. Therefore, a total of 41 incidences of complications/adverse events were recorded resulting in an overall complication rate of approximately 7.82% with 3.62% requiring a return to the operating room including those requiring manipulation.

**Table 2.** Complications/Adverse Events Incidence within Ninety Days of Index Surgery in XPERIENCE-Treated Primary TKA Cohort

Complication/adverse event description	Incidence # among 524 TKAs	Incidence rate
Superficial incisional SSI	1	0.19%
Organ/space SSI/early onset PJI	1	0.19%
Patients requiring incision and drainage for delayed wound healing and suture abscess without SSI diagnosis	3	0.57%
Patients with wound issues not requiring incision and drainage	12	2.29%
Arthrofibrosis requiring manipulation under anesthesia	10	1.90%
Arthrofibrosis necessitating arthroscopy	1	0.19%
Traumatic medial retinacular rupture/patellar subluxation	2	0.38%
Traumatic fall resulting in superficial wound disruption requiring closure	1	0.19%
Pulmonary embolism/DVT	2	0.38%
Temporary (partial) foot drop	3	0.57%
Peri-prosthetic fracture (non-operative care)	4	0.76%
Hospital re-admission for syncope	1	0.19%
Overall complication	41	7.82%

TKA = total knee arthroplasty; SSI = surgical site infection; PJI = peri-prosthetic joint infection; DVT = deep vein thrombosis.

## Discussion

The number of TKAs in the United States is expected to increase significantly, with some reports estimating exponential increases of 401% between 2014 and 2040^2^ while others estimating more moderate increases of 143% by 2050 compared with 2012^32^ or 85% by 2030 compared with 2014.^33^ There are several factors that have spurred the shift of primary TKAs from inpatient to outpatient settings in recent years.^34,35^ The peer-reviewed scientific literature has multiple publications comparing complication and re-admission rates in outpatient/ambulatory versus inpatient TKAs. These studies are a combination of retrospective, prospective, and meta-analysis evaluations, with varying patient screening methods, varying peri-operative protocols, varying degrees of matched or unmatched cohorts, and even varying definitions of outpatient surgery. Some have demonstrated no differences in complications or re-admission rates^9,14,36^ whereas others have demonstrated lower 30- and 90-day re-admission or complication rates in outpatient TKAs^15,37^ and yet others have demonstrated higher risks of complications in outpatient compared with inpatient settings^12,38,39^ thus making conclusive assessments of complication risks and SSI rates for outpatient TKAs challenging.

In general, SSI accounts for 20% of all hospital acquired infections, and is associated with substantial morbidity, a two- to 11-fold increase in the risk of mortality, and an estimated annual cost range of $3.5 to 10 billion in the United States.^40^ Surgical site infection incidence rates are reported at approximately 1% to 2%, and without a reduction in current SSI rates, a 14% increase in complex SSI is anticipated between 2020 and 2030 after hip and knee arthroplasty with a projected total burden of 77,654 SSIs.^17,18,41^ Eighty percent of infections in humans including SSI/PJI can be attributable to a microbial biofilm matrix.^21,22^ Bacteria protected within the biofilm matrix are far more resistant to antibiotic agents, antimicrobial agents, antiseptic agents, and host immune defenses thus, preventing biofilm formation is critical to preventing SSI.^23–27^ Multiple strategies continue to be utilized to address prevention of SSI/ PJI in total joint arthroplasty.^31,40^ The utilization of intra-operative antimicrobial irrigation solutions to decrease contaminating bioburden in the surgical site continues to be advocated.^28–31,41,42^

From 2016 through 2020, Edgewater ASC instituted multitiered infection prevention protocols which encompassed rigorous facility/operating room decontamination, pre-operative skin and nares decolonization protocols, and negative pressure wound dressings. Edgewater ASC instituted the intra-operative use of XP in May 2021 to clean and remove debris, including microorganisms from the surgical wound space. XPERIENCE was designed based on a patented technology platform called XBIO^TM^ that utilizes a material science approach to prevent the formation of neobiofilm as well as treat mature biofilm. The technology combines a buffered pH modifier and surfactant that work in concert to deconstruct the biofilm matrix by chelating metal ions from the EPS matrix, bringing the polysaccharides and microbes into solution and inducing bacterial cell lysis due to the surfactant and high osmolarity environment.^43^ The buffered pH modifier components of XP, namely citric acid and sodium citrate, provide unique advantages. Citric acid is a natural metabolism product, which is biocompatible and antimicrobial.^44^ In solution they are viewed as functional excipients and used widely in multiple industry applications to suppress microbial growth.

Because the novel irrigant does not require a soak time or a rinse after use, its intra-operative use does not increase operating time. Additionally, the no-rinse feature facilitates residual XP in the incision space to continue its prophylactic activity for an extended period after closure.^45^ In in vitro studies, XP exhibited 6 log or greater reduction in five minutes against planktonic forms of *Pseudomonas aeruginosa* and *Staphylococcus aureus*, known causative agents of SSI/PJI. It also exhibited increasing log reduction capability over time (5 minutes to 5 hours) against SSI/PJI causing bacteria established when present in a biofilm.^45^ The novel irrigant exhibited equivalent, or better antimicrobial inhibitory and eradication activity against these and other microorganisms in planktonic or mature biofilm form when compared to betadine solution containing 10% w/v povidone iodine (PI).^46^ Organizations such as the CDC and World Health Organization have addressed PI as an irrigant prior to wound closure.^47,48^ In contrast to XP, a soak time in the surgical cavity is recommended for dilute PI followed by an extensive rinse with saline.^49^ Although PI is administered manually, XP can be administered manually or via pulse lavage and is compatible with most pulse lavage systems on the market.

This retrospective study evaluated the impact of XP on SSI/early onset PJI incidence in primary TKA performed in an ASC setting. To our knowledge, this is the first-real world evidence report addressing the efficacy of this irrigant in an in vivo ASC setting. Notable findings were that there was one diagnosed organ/space SSIs/early onset PJI within 90 days of index surgery and one superficial incisional SSI within 30 days of index surgery in 524 primary TKAs. There was no diagnosis of deep incisional SSI in the cohort. The sole PJI diagnosed within 90 days of index surgery was unmistakably associated with an exogenous group A streptococcal upper respiratory tract pharyngitis infection that occurred several days after post-index surgery associated with an identified exogenous exposure. It can be reasonably assumed that the single incidence of early onset PJI in this cohort was not attributable to failure of intra-operative infection prevention regimes.

The variability in the definition and timelines for diagnosing SSI, variability in SSI incidence rates and complication/adverse event rates in outpatient TKAs and the varying definitions of “outpatient” joint arthroplasty cited in the literature, makes a robust comparative evaluation of the current retrospective clinical study to historical ASC data, challenging. A non-expansive review of the literature determined that infection-related events in this retrospective study were consistently below or at the lower range of historical reported rates (Table 1). Historical rates of SSI/PJI post primary TKA range between 0.15% to more than 2%. Thus the 0.19% superficial incisional SSI rate and 0.19% PJI rate in this retrospective study indicated statistically significant efficacy of XP in decreasing SSI/PJI at the higher reported rates.

Re-admissions rates because of infections in primary TKA are reported at approximately 1.5%.^50^ The two re-admissions attributable to SSI post-90 days of index surgery in this study at 0.38% are comparable or better than the most favorable literature reported re-admission rates. The overall complication rate of 7.82% in this retrospective study was also well within the historical reported complication rate ranges (5.4%–16%) in outpatient TKAs.^13^

## Conclusions

This study evaluated the effects of XP on SSI/PJI occurrence, SSI-related re-admissions, complications, and adverse events among 524 TKAs performed at a single ASC facility by a single orthopedic surgeon. In this retrospective clinical study, the novel irrigant exhibited substantial efficacy at decreasing SSI/early onset PJI incidence when compared with most historically reported infection rates. Unlike other commonly used intra-operative irrigants such as PI that require a rinse, the no-rinse feature of XP conferred added advantages of usability and potentially extended the activity in the surgical site space after surgical incision closure. This study warrants further investigations of XP in comparison to other intra-operative irrigants, in robustly designed, prospective, multicenter clinical trials in inpatient and outpatient joint replacement surgeries to evaluate the definitive utility of this novel irrigant to lessen the overall burden of SSI/PJI and improve patient outcomes.

### Limitations

This retrospective analysis was conducted in a small cohort of 524 primary TKA surgeries in a single ASC facility conducted by a single surgeon and lacked a comparative evaluation to a control cohort not treated with XP. Given the variable range of historically reported SSI/PJI rates, a definitive finding of the efficacy of the novel irrigant in decreasing SSI/PJIs in this retrospective clinical study to enable extrapolation to settings with different surgical protocols or patient profiles is challenging.
